# “Why must I get an infection, especially after surgery?” opportunities for patient engagement in infection care

**DOI:** 10.1017/ash.2025.10062

**Published:** 2025-09-17

**Authors:** Oluchi Nneka Mbamalu, Estelle van Tonder, Ebruphiyo Ruth Useh, Bongeka Mfeketo, Okuhle Pretty Mbengo, Adam Boutall, Timothy Pennel, Lebohang Moloi, Salome Maswime, Esmita Charani, Marc Mendelson

**Affiliations:** 1 Division of Infectious Diseases and HIV Medicine, Department of Medicine, Groote Schuur Hospital, University of Cape Town, Cape Town, South Africa; 2 Global Surgery Division, Department of Surgery, Groote Schuur Hospital, University of Cape Town, Cape Town, South Africa; 3 Medical School, Department of Medicine, University of Cape Town, Cape Town, South Africa; 4 Institute for Life Course Health Research, Department of Global Health, Stellenbosch University, Cape Town, South Africa; 5 Synergy Biomed Research Institute, East London, South Africa; 6 AFDA Film School, Cape Town, South Africa; 7 Colorectal Unit, Groote Schuur Hospital, University of Cape Town, Cape Town, South Africa; 8 Chris Barnard Division of Cardiothoracic Surgery, Groote Schuur Hospital, University of Cape Town, Cape Town, South Africa; 9 Acute Care Surgery Division, Department of Surgery, Groote Schuur Hospital, University of Cape Town, Cape Town, South Africa; 10 Faculty of Health and Life Sciences, University of Liverpool, Liverpool, United Kingdom

## Abstract

**Objective:**

We explored the awareness, perspectives, and experiences of patients and healthcare providers (HCPs) regarding opportunities for surgical patients to engage in infection care.

**Design:**

Qualitative research design.

**Setting:**

Tertiary academic hospital, Cape Town, South Africa.

**Participants:**

79 patients and 22 carers were observed during interactions with HCPs, and interviews were conducted with 19 patients, 3 carers, and 8 HCPs.

**Methods:**

Data were collected through nonparticipant observations of surgical wound care and patient consultations, and in-depth semi-structured interviews using data collection guides. Data were coded inductively, aided by NVivo software (version 14) and themes identified.

**Findings:**

Three main themes emerged from the data: patient’s healthcare knowledge following surgery, patients’ experiences of surgical site infection, and patients’ experiences as users of the healthcare system. Care discussions were largely driven by the doctor, and focused on the patient’s condition, associated diagnostic tests and medicines, and wound care. Patients’ contributions were mostly passive, as recipients of information and respondents to doctors’ questions. Most patients expected HCPs to initiate engagement and were unaware of healthcare-associated infections before developing one.

**Conclusions:**

Our data highlight a need to strengthen postdischarge wound care. The presentation and delivery of information in patient-facing and relatable formats can improve patient and carer understanding of surgical infection risks and care, and help support and advance patients’ roles as co-stakeholders in infection care for improved health outcomes.

## Introduction

Surgical site infections (SSIs) complicate surgical outcomes, particularly in low- and middle-income countries (LMICs)^1^. They contribute to mortality (up to 38% of postoperative deaths) and economic costs (additional costs from $174 to $29610 per patient).^2,3^ Ongoing efforts to prevent SSIs and improve antibiotic prescribing and use in African hospitals are on the increase, with focus on guideline implementation for infection prevention and control (IPC), antibiotic stewardship programs, healthcare worker training and capacity building, and infrastructure improvement.^4,5^ South Africa’s Antimicrobial Resistance (AMR) National Strategy Framework and facility-based antimicrobial stewardship programs (AMS) aim to improve antibiotic prescribing.^6,7^

With patients and families among the stakeholders involved in postoperative management of surgical wounds, their roles are crucial for surgical infection outcomes. Strategies to improve outcomes have, however, predominantly focused on healthcare provider (HCP) roles.^8^ Research has highlighted the missed opportunities and key roles for engaging surgical patients in infection care.^8,9^ Patient involvement in preventing or managing infections such as SSIs and patient engagement in AMS have been proposed as suitable additional strategies for infection care bundles.^8–11^

To participate in IPC and AMS, patients need an appreciation of the burden that AMR places on global healthcare systems, including worse outcomes, increase in length of hospital stay, and increased health expenses in LMICs, including Africa’s often-fragile healthcare systems.^12–14^ This research explored the experiences of patients and HCPs, identifying opportunities for patient and carer engagement in infection-related care.

## Methods

### Study design

Qualitative research design comprising nonparticipant ethnographic observations and in-depth participant interviews. Data were collected using modified versions of previously developed data collection guides (Appendices A—C).^15^ Study participation was voluntary; participants could decline participation, with no risk of prejudice. ^16^

### Study setting and participants

The study was conducted in the outpatient departments of the acute care (Specialty A), colorectal (Specialty B), and cardiovascular and cardiothoracic (Specialty C) surgery specialties at Groote Schuur Hospital, a tertiary academic public referral hospital in Cape Town, South Africa. Participants were adult patients, or their carers, who visited the specialties for surgery-related care or follow-up, and HCPs involved in their care.

### Patient and public involvement

All patients, carers, and HCPs present during the outpatient case who had consented to participation were included in the ethnographic observations. Semi-structured interviews were conducted with patients who had a confirmed SSI and their carers (when available), following receipt of written informed consent. All participant information was anonymized prior to analysis and for presenting study findings.

### Data collection

Data were collected from May 2022 to March 2023, until saturation was reached. Participants were invited to participate when they visited the selected out patient clinics. Participants could decline participation at any time, even after initially approving. While observations were not envisaged to alter patient care, researchers refrained from observation when they felt a particular patient/HCP would prefer so or when they thought it might negatively impact patient care; for instance, if there was limited space in the observation area. Observations were documented in field notes with identifiers anonymized.

Interviews took place at the participant’s convenience, away from clinical duties or care in either English, Afrikaans, IsiXhosa, or a mixture of these. Semi-structured interview guides featuring open-ended questions allowed for follow-up of new ideas that emerged during the interview. Interviews were audio-recorded, transcribed verbatim, and identifiers anonymized for data presentation.

### Data analysis

Data from observations and interviews were coded using NVivo (version 14). Inductive coding facilitated pattern identification in the data, which were further streamlined into themes. Data collection using more than one method, reflexivity, and discussions of emerging themes ensured rigor. We employed Oben’s^16^ conceptual framework to explore patients’ experiences of illness and healthcare services, commencing when an individual starts moving along the care continuum until recognized as a “patient” at disease onset or presentation to a healthcare facility. Further along this pathway, the “patient” becomes a “user” on interacting with the healthcare system and HCPs, and utilizing healthcare services.

## Results

### Participant characteristics

Data collectors observed 79 patients and 22 carers for over 50 hours in total, during patient-HCP interactions (cases of wound dressing or consultations with surgeon) . From the patient cohort, interviews were conducted with 19 patients who said they had experienced an SSI, 3 carers, and 8 HCPs (Table 1). The most (15/19) of patients were referred from private doctors’ rooms, community healthcare centers, and district and regional healthcare facilities.

**Table 1.** Demographic details of participants interviewed according to gender, age, and care specialty

Category	S/no	Gender	Age	Specialty
Patients	1	Female	50–60	C
	2	Female	60–70	A
	3	Male	40–50	A
	4	Female	20–30	A
	5	Female	50–60	B
	6	Female	50–60	A
	7	Male	20–30	B
	8	Male	40–50	B
	9	Male	30–40	A
	10	Female	60–70	A
	11	Female	40–50	A
	12	Female	40–50	B
	13	Male	50–60	B
	14	Female	30–40	A
	15	Male	50–60	A
	16	Female	30–40	A
	17	Male	40–50	A
	18	Female	20–30	A
	19	Female	30–40	A
Carers	1	Male	50–60	C
	3	Male	40–50	A
	17	Female	40–50	A
Healthcare providers	1	Female	50–60	A
	2	Female	50–60	A
	3	Female	50–60	A
	4	Female	50–60	B
	5	Female	50–60	B
	6	Female	30–40	C
	7	Male	30–40	C
	8	Male	30–40	C

Specialty A: acute care surgery; Specialty B: colorectal surgery; Specialty C: cardiovascular and thoracic surgery..

Thematic analysis of field notes and transcripts identified three main themes. Complementary quotes are referred to by the Quote Number (Appendix D).

### Patient’s healthcare knowledge following surgery

#### Gaps in the discharge pathway

Concerning patients’ postsurgery experiences, observations, and interviews highlighted limited awareness of how to differentiate normal wound healing compared to evolution of an SSI that is, some patients did not know what to look out for or how to care for their wounds (Q1, Q2).

SSIs reportedly occurred postdischarge. Patients who delayed presenting for SSI care mentioned not being aware of the extent of the complication. Occasionally, patients reported not receiving discharge information or guidance. HCPs who reflected on this clarified that patients received discharge information, including wound care information and sites for follow-up care; however, challenges were noted during follow-up at some primary healthcare centers after discharge (Q3, Q4).

#### Discharge information guidance and facilitators

A patient’s social circumstances impacted on wound care. Patients who had an accompanying carer present when discharge information was provided, handled self and wound care better, as care guidance was more likely to be remembered (Q5, Q6). Whilst some participants recalled being provided with discharge information, patient feedback highlighted the detrimental effect of pain and exhaustion on information recall.

The data point to a lack of ownership regarding responsibility for providing wound care and postoperative care information on discharge from the hospital. Patients could not identify who was supposed to have provided them with discharge information, particularly on wound care. There was no consensus on the best time or platform for patient engagement on postdischarge infection care; participants had varied preferences for preadmission, inpatient stay, and during discharge. They were equally neutral on how the information should be provided, that is, verbal, written, or accessible digital formats.

#### Lack of awareness of healthcare-associated infections

Almost all interviewed patients had SSI as a postoperative complication before or around the study time. Patients were generally unaware of HCAI risks and did not hitherto consider infection risk among potential complications of hospitalization. The hospital was seen as a place of minimal infection risk given its clean appearance (Q7 – Q11). Participant’s knowledge and awareness of SSIs generally occurred after the participant or someone close to them had suffered an SSI, which increased their dedication to infection prevention measures (Q7 – Q11).

While some participants perceived patient engagement and awareness of infection risks as essential to prevent infections, others preferred to be unaware of such risks, reasoning that it was not inevitable that it would be helpful (Q12, Q13). Patients who recalled infection risk information were more aware of HCAI risks (Q11, Q14).

None of the interviewed patients expressed awareness regarding AMR when first mentioned. On further questioning and with more in-depth discussions, participants recalled infections suffered by others, that seemed to describe resistance, as they referred to “*infection(s) that could not be treated*.”

Participants did not report receiving AMR information from HCPs. In interviews with nurses, they reflected on not being privy to patients’ AMR laboratory results unless such was communicated by the doctor. As patients’ culture results were not enclosed in their folders, nurses felt incapable of counseling on antimicrobial-resistant infection risks and care unless the doctor had informed them about the resistance profile of the bacterium causing an infected wound. When not communicated, nurses assumed the wound infection was susceptible and not resistant to treatment, which influenced their AMR communication or lack thereof.

### Patients’ experiences of surgical site infection

Infections were noted to negatively affect well-being. Patients’ experiences of the interval between discharge and SSI development resulted in a sense of diminishing quality of life and inability to partake in meaningful personal experiences. Examples were an inability to breastfeed or bond with their new-born baby, inability to relate with social contacts, or return to presurgery lifestyle, dependence on others, and loneliness (Q15, Q16). These negative experiences were exacerbated by limited psychosocial support, especially for those with pivotal events (Q17 – Q19).

### Patients’ experiences as users of the healthcare system

#### Patients’ engagement experiences with healthcare providers

Intentional HCP engagements with patients were positively perceived and deemed empowering. As users of healthcare services, feeling heard encouraged patients to discuss issues with the HCP and improved care experiences (Q20 – Q23). In cases where this communication was lacking, patients reported poor healthcare experiences.

Patients who asked questions received additional information on care dynamics, compared to those who did not. Patients generally assumed that HCPs would initiate engagement where needed and consequently, did not think they had to proactively initiate communication. This was underscored during interviews when a participant explained why she did not ask questions about her care (Q25)? In some cases, patients did not know what to ask or how to initiate communication regarding their care. Some reported discussions with a HCP or family as helpful guidance, which empowered them to ask questions about their care (Q26, Q27).

**Figure 1.**
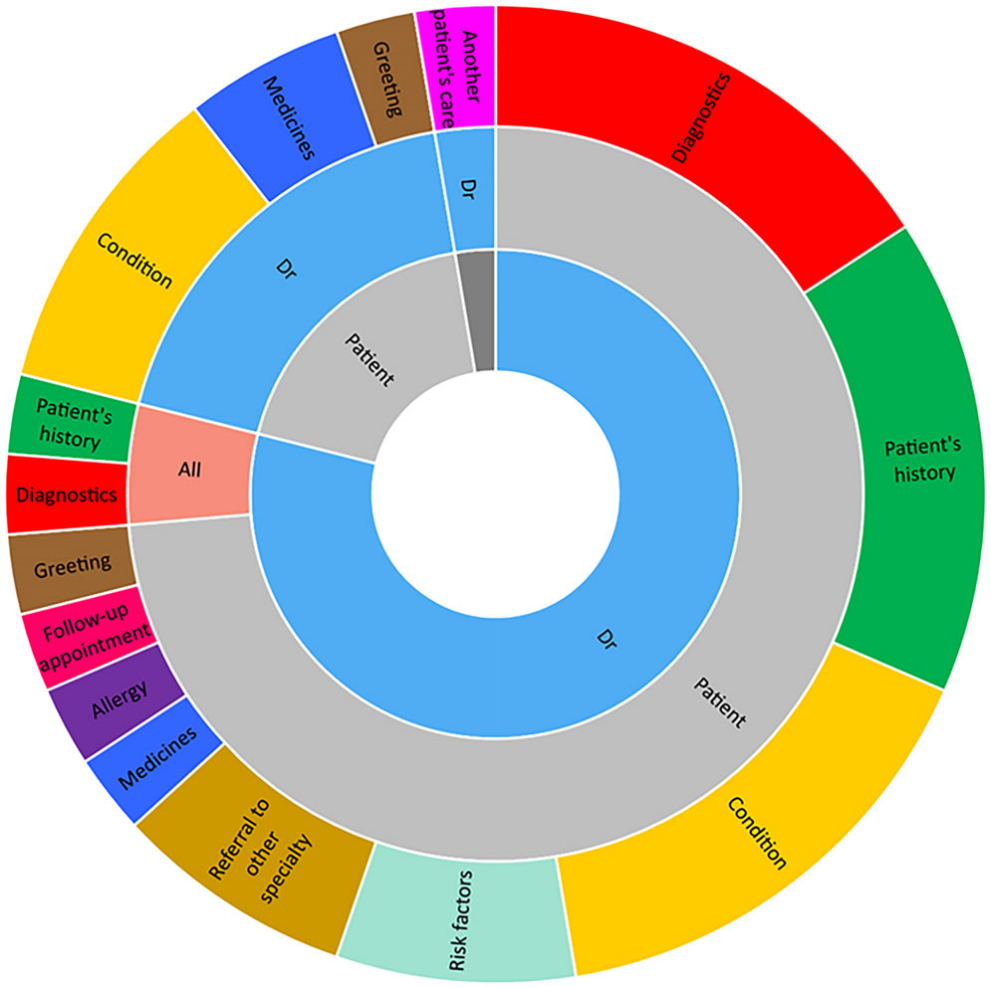
Care discussion initiators, recipients, and topics for a patient during consultation with a doctor in specialty A. This Figure illustrates the discussions which were observed to mostly be initiated by the doctor (inner ring), with the patient generally a recipient of information or respondent to questions (middle ring) on the various topics (outer ring). Discussions initiated by the patient were related to their care and clarification regarding medicine use for their condition..

In Figure 1, an example of patient-HCP communication during consultation with a doctor is presented; infection-related communication was limited except when a patient’s wound was infected and in the wound dressing clinic, focused predominantly on wound care.

#### Poor healthcare provider-patient engagement and experiences

Notable barriers to patients engaging in their infection-related care discussions included fear, patient exclusion from care discussions, and previous negative healthcare experiences such as surgical complications and evasive responses (Q27 – Q31). Such barriers influenced patient presentation for hospitalization and treatment (Q32).

Some patients mentioned that initiating engagement may be misconstrued as unhelpful interruption. HCPs considered this and acknowledged their role as patient and care advocates, and the crucial nature of patient engagement for care progress (Q33, Q34).

## Discussion

In this research, we captured the views of patients carers, and HCPs on surgery-related infection care and explored opportunities for patient engagement. Click or tap here to enter text. Our study findings underscore how SSIs impact patient and family well-being and quality of life, as noted in previous studies.^17–19^

Our findings revealed a dynamic journey. According to Oben’s framework, the patient begins as an individual in relatively good health postsurgery, prior to SSI development. The patient is nested within a social context where they are supported by family and informal carers. At SSI occurrence, the patient experience shifts dramatically as they move from uncomplicated postsurgery recovery to one requiring SSI treatment. During this time, the patient faces a stage marked by diminished quality of life due to negative effect by the SSI, and a heightened need for psychosocial support. Subsequently, the patient, continuing this journey within the healthcare system, is recognized as a service user, retaining their initial identity as an individual seeking healthcare—like the condition in which they had initially approached the health facility prior to surgery.

We noted gaps in patients’ knowledge of postsurgical self-care, HCAI awareness, and psychosocial support. Several patients were poorly equipped to assess progress of surgical wound healing, following discharge.^17^ As such, infection care needs and conditions may go unrecognized by patients who, away from the hospital, manage discrete infection-care risks and care routines as they arise. Inadequate information and communication related to patient care can result in limitations to patients’ navigation of the healthcare journey,^18,9^ as noted in this study. However, patients provided with discharge information fared better with postop wound care.^18^ These findings underscore a need to improve discharge planning as a tool to reduce infection complications as low-level and poor quality of discharge education is known to decrease patient readiness for hospital discharge.^19^ Discharge care information should be provided at an appropriate time for patient engagement, which may differ case by case.^9^ Subsequently, information recall by accompanying carers stresses carer roles in patient care and the need for their inclusion in patient-centered infection care programs.^20,21^

It is also evident that shortfalls in patient education—whether due to insufficient instruction, ineffective delivery, or patient’s inability to engage at the time—are associated with adverse patient outcomes. Research highlights systemic challenges, such as unclear accountability for education, contributing significantly to this problem. For instance, Lau et al. (2016) and de Silva (2011) noted that organizational ambiguity and structural barriers in primary care often lead to an evidence-to-practice gap, affecting both patient engagement and care continuity,^22^ and patient empowerment through education is effective only when established processes exist to verify understanding and ensure accountability.^22,23^ Additionally, Dineen-Griffin et al. (2019) emphasized the need for systematic approaches that go beyond mere information delivery to incorporate structured feedback, ultimately enhancing self-management.^24^ These studies collectively reinforce the importance of distinguishing between a lack of education and suboptimal educational delivery, each of which requires distinct strategies to improve patient outcomes.

Another observation, through our interviews, was the participants’ limited awareness of HCAIs such as SSI and AMR. Most participants were referred from one or more facilities before presentation at the study site, given the site’s function as a tertiary facility. Participant’s limited AMR awareness, even after such referrals, highlights an early gap in patient engagement across the healthcare continuum.

Although previous research has also documented patients’ insufficient knowledge and misconceptions of HCAI,^25–27^ our findings, together with a nonrandomized survey conducted at this study site, indicated that patients may recognize specific infection care terms when described.^10^ Other studies among patients in South Africa highlight issues of comprehension related to antibiotics and AMR when these terms do not exist in the native language.^28–30^ These findings support the need to address language challenges when communicating infection care and AMR information for patient and public audiences.^31–33^

Generally, continuity of wound care postdischarge emerged as another critical issue, most patients having been referred to the primary healthcare facility nearest to their residence. However, some patients reported poor experiences and felt their surgical wound condition worsened at these facilities, resulting in their preference for care and follow-up at the tertiary care level study site. Such care continuity at tertiary care level can obstruct referral pathways. However, we acknowledge that patients interviewed in our study had complicated wounds, possibly needing specialist wound-care attention, precipitating their referral to the study site.

The experiences of our interviewed patients traversed multiple domains, which overlapped most of the nine domains reported by Kemp et al.^20^ These support similarities in domains of healthcare experiences across different settings (eg, communication with HCPs regarding medicines, staff responsiveness, cleanliness of healthcare facility, discharge information) and the importance of such domains for evaluating patient experiences. With AMR often unknown to patients, developing patient-facing materials to raise AMR awareness among patients, carers, and the public would be beneficial. In line with COVID-19 experiences, AMR-related data could be presented with charts and relatable graphics in patient-facing and community-facing formats. Short- and medium-term strategies, supported by universities, can enhance HCP–patient communication in both hospital and ambulatory settings. Furthermore, exploring experiences in surgical infection care shows that professionals should initiate engagement, and patients need support to ask questions and get involved. These findings are timely and align with ongoing national and global efforts to strengthen healthcare systems since the COVID-19 pandemic,^34^ emphasizing the role of informed and involved patients in achieving better care outcomes.

This study benefits from several strengths, including the use of structured data collection guides, methodological triangulation, and involvement of diverse data collectors, including members of the public, which enriched the data with varied perspectives. It also employed local languages, enhancing inclusivity. Our data may have been biased by participant’s recall, and the researchers’ presence may have influenced participant responses. Additionally, purposive sampling of individuals who reported SSIs may limit the generalizability of findings.

Despite this, the study offers valuable insights with practical implications. It underscores the need for care managers to strengthen infection safety nets beyond hospital discharge, which may involve partnerships to improve accessibility. Through the healthcare pathways of various facilities, patient management teams need to factor in patient education on HCAIs to promote patient engagement in infection prevention.

## Conclusions

This study highlights an opportunity for care managers to consider infection care safety nets for patients in the continuum of care, especially outside the hospital. These can involve partnerships to make postdischarge wound care more accessible.

Strengthening infection safety nets beyond hospital discharge and improving public-facing communication on AMR are essential steps toward better engagement and outcomes. The, experiences and feedback of patients without infection complications could be used, along with the experiences reported in our study, to inform quality improvement initiatives for patient care.

## Supporting information

10.1017/ash.2025.10062.sm001 Mbamalu et al. supplementary material 1 Mbamalu et al. supplementary material

10.1017/ash.2025.10062.sm002 Mbamalu et al. supplementary material 2 Mbamalu et al. supplementary material

10.1017/ash.2025.10062.sm003 Mbamalu et al. supplementary material 3 Mbamalu et al. supplementary material

10.1017/ash.2025.10062.sm004 Mbamalu et al. supplementary material 4 Mbamalu et al. supplementary material

## Supplementary material

The supplementary material for this article can be found at https://doi.org/10.1017/ash.2025.10062.
