# Supplementary material for: “Why must I get an infection, especially after surgery?” opportunities for patient engagement in infection care
Source: Antimicrob Steward Healthc Epidemiol. 2025 Sep 17;5(1):e223. doi: 10.1017/ash.2025.10062 (PMC12451813; doi:10.1017/ash.2025.10062)
Supplement: Mbamalu et al. supplementary material 1 — Mbamalu et al. supplementary material [file S2732494X25100624sup001.pdf]

Research study: Infection prevention and control and antimicrobial stewardship in the surgical out-patient pathway: opportunity for patient engagement?

Table 1: Description of data to be collected during ethnographic observations

**Episode of Observation**	**Surgical out-patient department (OPD)**	**Consulting rooms**	**Pharmacy**	**Shadowing individuals**
**Data to be collected**	Duration<br><br>The time of day<br><br>A general description of the out-patient department including the people present and the activities taking place<br><br>The patterns of activity	Duration<br><br>People in attendance<br><br>Who led the consultation?<br><br>What was discussed – who lead the discussions, who contributed?<br><br>What tools or methods were used to engage the patient, e.g., . descriptions, graphics, smart phones, etc.?<br><br>What tasks were identified?<br><br>Who was responsible for carrying out tasks?<br><br>What interactions there were with patients<br><br>What interactions there were with other healthcare professionals?<br><br>What each person did during the consultation, i.e., what they contributed or if not contributing, what they were doing<br><br>Any emotions expressed or felt<br><br>Observer contribution, if any, to the activities, e.g., pulling curtains, getting gloves or hand sanitiser for consultant<br><br>Any disruptions to the activity	Duration<br><br>Who attended?<br><br>What was discussed?<br><br>Who led the discussion?<br><br>Who contributed to the discussion?<br><br>What, if any, data was used or presented?<br><br>Who presented the data?<br><br>Any emotions expressed or felt?	Duration<br><br>Type of activity, e.g., patient journey in the surgical OPD pathway<br><br>Any dialogue between observer and participant<br><br>Any disruptions to the activity<br><br>The interactions of the healthcare professional with patients and other members of staff<br><br>Places visited<br><br>Tools used by patient, e.g., smart phones<br><br>Observer contribution, if any, to the events taking place e.g., helping the pharmacist/ doctors with any required items
