# Supplementary material for: “Why must I get an infection, especially after surgery?” opportunities for patient engagement in infection care
Source: Antimicrob Steward Healthc Epidemiol. 2025 Sep 17;5(1):e223. doi: 10.1017/ash.2025.10062 (PMC12451813; doi:10.1017/ash.2025.10062)
Supplement: Mbamalu et al. supplementary material 2 — Mbamalu et al. supplementary material [file S2732494X25100624sup002.pdf]

NRF Study: Infection prevention and control and antimicrobial stewardship in the surgical out-patient pathway – opportunity for patient engagement?

Interview Guide: Patient/Patient Carer/Public

Category of enquiry	Sample questions
**Opening questions:**	Can you tell me why you (or your patient or family member) are/were in the hospital today/on ______?<br><br>Are/were you accompanied by anyone? If so, who?
**Patient/carer involvement in SSI/IPC/AMS prior to surgery**	1. Did anyone tell you what to expect before surgery?<br><br>2. Has anyone spoken to you about surgical wound infection?<br>*If* ***Yes***, what have they said?<br>*If* ***No***, what does surgical wound infection mean to you?<br><br>3. Were you informed of any measures that will help to reduce surgical wound infections?<br>- *If* ***Yes***, can you remember what these were?<br><br>4. Have you heard about *antimicrobial resistance or antibiotic resistance*?<br>*If* ***Yes***, what does it mean to you?<br>*If* ***No***, what do you think this term refers to? What does it mean to you?<br><br>5. What do you think you could do as a patient to manage or prevent infection whilst in hospital?<br><br>6. Do you think your family / carers / hospital visitors played a role in your infection management and prevention?<br>Did someone discuss this with you prior to surgery?<br><br>7. Were you prescribed any medicine to treat infection **before** surgery? If so:<br><br>- Did someone explain how you were to take them?<br>- Did someone explain the reason for prescribing them?<br>- Did someone explain what type of medicines they were?<br>Did someone explain the infection risks when taking them incorrectly?<br>- Did you take them as directed? *If* ***Yes***, why? *If* ***No***, why?
**Patient/carer involvement in SSI/IPC/AMS after surgery**	8. Did anyone tell you what to expect after surgery?<br><br>*If* ***Yes***, did this include anything about surgical wound infections?<br>*If* ***No***, can you tell me what you expected?<br><br>9. Did this include information on infections that are difficult to treat or resistant to antibiotics/antimicrobial medicines?<br>10. Did this include information on appropriate use of medicines for infection?

	11. **Surgical wound care after discharge**<br>- Before discharge, did you receive any information on how to care for your surgical wound?<br>- What was your experience of (surgical) wound care after discharge?<br><br>12. When did your wound become infected (in hospital or after discharge)?<br>- How did you know it was infected?<br>- How did you feel when you thought/knew it was infected?<br>- Why do you think it became infected?<br>- Did you seek advice for infection care (of your surgical wound)?<br>- *If so*, who did you seek advice from?<br>- When did you seek advice from this person?<br>- Why did you seek advice from this person?<br><br>- *If patient did not seek advice for care of surgical wound,* Can you tell me why?<br><br>13. Do you think you (as the patient) had a role or responsibility in your infection care **after surgery**? This could include taking your medicines as prescribed and keeping your wound clean and sterile<br><br>14. Were you prescribed medicines to treat an infection **after** surgery?<br>If ***Yes***,<br>- Did someone explain how you were to take them?<br>- Did someone explain the reason for prescribing them?<br>- Did someone explain what type of medicines they were?<br>- Did someone explain the infection risks when taking them incorrectly? Did you take them as directed?<br><br>- *If* ***Yes***, why? *If* ***No***, why?
**Current patient/public roles in IPC/AMS and influencing factors**	15. What do you think of the way that patients are engaged in infection care?<br>Can this be improved? *If* ***Yes****,* How?<br>Is there anything that healthcare workers or patients themselves can do to improve engagement in infection care?<br>What do you think patients can do to assist in their infection care?<br><br>16. To what extent do you think patients are aware of antimicrobial resistance / antimicrobial resistant infection, or antibiotic resistance / antibiotic resistant infection?<br><br>17. What did you do to manage or prevent infection (in and out of hospital) and was it successful?

	18. Along the whole surgical pathway, where do you think is/are the most important components) to engage the patient:<br>- in infection prevention and control (IPC)?<br>- in responsible use of medicines for infection?<br>- both<br><br>19. What specific activities can the patient be engaged in, with respect to infection prevention and care or appropriate use of medicines to reduce the risk of infection?<br>- How would you, as a patient like to be engaged in infection care?<br>- What information do you feel could have been communicated better?<br>- What do you think is the most effective way to communicate infection care and appropriate medicine use? (For example a pamphlet when you are admitted to hospital, a poster in your hospital room, a conversation with a health professional, a website or social media, an infection management mobile app, etc)<br><br>20. Did you provide any feedback (to the health care team or anyone else) on how you were engaged in infection care or appropriate use of medicines?<br>*If* ***Yes***, would you like to share this?
**Patient perceptions of roles and responsibilities related to AMR, SSI and AMS**	21. **Before** and/or **after** surgery, did some members of the health care team (e.g., your doctor, nurses, pharmacist, etc) engage you in infection care and prevention or appropriate use of medicines for infection?<br>- If so, who were these and what impact do you think they made in your care?<br>- Is there anyone among them whose role you valued the most?<br>- (*If it applies*) Overall, what do you think had the most influence on your engagement?<br>- Why do you think so?<br><br>22. How easy was it for you to ask questions about infection care and prevention or use of medicines for infection, or to obtain information elsewhere?<br>- Were there any:<br>- facilitators that made this easier?<br>barriers/challenges (if so, can you mention these)?
**Final question**	23. Is there anything you would like to add to what we have discussed?

Thank you for taking the time to participate in this research.

<u>Additional Information</u>

Participant:
Specialty:
Age of patient:
Gender of patient/patient carer
