# Supplementary material for: “Why must I get an infection, especially after surgery?” opportunities for patient engagement in infection care
Source: Antimicrob Steward Healthc Epidemiol. 2025 Sep 17;5(1):e223. doi: 10.1017/ash.2025.10062 (PMC12451813; doi:10.1017/ash.2025.10062)
Supplement: Mbamalu et al. supplementary material 3 — Mbamalu et al. supplementary material [file S2732494X25100624sup003.pdf]

NRF Study: Infection prevention and control and antimicrobial stewardship in the surgical out-patient pathway – opportunity for patient engagement?

Interview Guide: Health Care Worker

Research study: Infection prevention and control (IPC) and antimicrobial stewardship (AMS) in the surgical out-patient pathway: opportunity for patient engagement?

**Category of enquiry**	**Sample questions**
**Opening question**	Can you tell me what your role is, in patient care, within your team?<br><br>In your team, do you assist in pre-op assessment of patients? Can you tell me what this involves?<br><br>*If not mentioned,* Does this include provision of information on infection prevention and control (IPC) and/or appropriate use of antimicrobials?
**Perceived HCW roles and responsibilities as related to patient engagement in infection care/AMS**	1. Do you think some other members of your team have a role in patient engagement in infection care/AMS? If so, who are these and what role(s) do you think they have?<br>- Is there anyone among them whose role is the most important or direct when it comes to engaging the patient in infection care or appropriate use of antimicrobial?<br>- What do you think has the most influence on patient engagement in infection care/appropriate use of antimicrobials in your workplace?<br>- Why do you think so?<br><br>2. From your observations and experience, does the patient have a role to play in infection care/AMS/prevention of SSI?<br>*If* ***Yes,***<br>- What role(s) is/are this/these?<br>- How do they come to know of this/these role(s)?<br>- Who explains the roles to them?<br>- How does this work in practice? In your experience, how have patients been engaged in or participated in infection care / AMS?
**Current patient / public roles in IPC/AMS and influencing factors**	3. How easy is it to engage your patients in infection care or appropriate use of antimicrobials?<br><br>4. What factors influence whether you or other members in your team engage a patient in infection care or appropriate use of antimicrobial?<br>- Are there any:<br>- facilitators that make this easier?<br>- any barriers (if so, can you mention these)?<br><br>5. What do you think of the way that patients are engaged in infection care and/or appropriate antimicrobial use?<br>Can this be improved? If so, how?<br>Whose responsibility (if any) needs to be worked on for improvement – the health care worker's or the patient's?

	6. Do the public have a role to play in patient engagement for infection care or appropriate antimicrobial use?<br>*If **Yes***, what is this role?<br>*If **Not***, why do you think so?
**Patient roles and responsibilities as related to AMR, SSI and AMS**	7. To what extent do you think patients are aware of antimicrobial resistance or antimicrobial resistant infection?<br><br>8. Do you think the patient has a role in preventing or managing SSI?<br><br>9. Along the whole surgical pathway, where do you think is / are the most important components) to engage the patient:<br>- in IPC?<br>- in appropriate use of antimicrobials?
**Data and indicators on patient engagement in IPC / AMS**	10. Are you aware of any **policies or guidelines**, in your specialty / workplace or elsewhere, for engaging the patient (***patient** in general or **surgical patient** specifically)* in IPC or appropriate antimicrobial use?<br>- If ***Yes***, how effective are these?<br>- If ***No***, why?<br><br>11. Are you aware of any data or indicators (e.g., patient satisfaction ratings, HCW performance feedback surveys or ratings by patients, etc) for assessing patient engagement in IPC appropriate antimicrobial use within your specialty or in the hospital?<br><br>12. With respect to patient engagement in infection care, is there any specific type of data that you would like to have available – at specialty, institutional or other organisational level? If **Yes**, why this/these specific data?<br><br>13. Are you aware of any patient safety committees within your specialty / the hospital?<br>a. Who is represented on the committee?<br>b. How often does the committee meet?<br>c. Is there a patient/ patient carer/patient advocate on the committee?<br>d. Is information from the meetings of this committee available to other teams? To what degree?
**Final question**	14. Is there anything you would like to add to what we have discussed?

Thank you for taking the time to participate in this research.

<u>Additional Information</u>

Participant:
Specialty:
Age:
Gender:
Years in practice:
