# Supplementary material for: “Why must I get an infection, especially after surgery?” opportunities for patient engagement in infection care
Source: Antimicrob Steward Healthc Epidemiol. 2025 Sep 17;5(1):e223. doi: 10.1017/ash.2025.10062 (PMC12451813; doi:10.1017/ash.2025.10062)
Supplement: Mbamalu et al. supplementary material 4 — Mbamalu et al. supplementary material [file S2732494X25100624sup004.docx]

**Appendix D: Participant Quotes**

| Quote # | Details |
| --- | --- |
| Q1 | *I do not know how the wound is supposed to look. I do not know if I can show you, but the wound looks worse now than it did when I was in hospital.*  *(Patient 11, Specialty B)* |
| Q2 | *When I left, after my operation, I had a dressing, and they said, “Do not touch it, until you come back.” So, there is that, but then they did not necessarily say, “Do not get it wet …”.*  *(Patient 15, Specialty A)* |
| Q3 | *I have come across feedback about health education not being given on discharge and that is the important part for the patient, to give it out when you do a discharge. You cannot just tell the patient, “Take your tablets as prescribed and off you go”, but that patient has got a wound. You must tell that patient, “This is what you look for”, “This is how you do it”, “This is where you must go on the day”, “This is what you need to dress that wound”, “Your day hospital* [primary healthcare facility] *is your first line if you do not have dressings”. The problem also lies at the day hospitals; they go for help at the day hospitals, but they do not get that help.*  *(HCP 1, Nurse)* |
| Q4 | *So, what we found out, by the time we would discharge the patient, doctor would say, “Okay, follow up at the day hospital* [primary healthcare facility]*.” In two to three weeks’ time, the patient would come back far worse than when we had discharged him. It is not nice for us to restart* [follow up] *with that patient, whereas we let them off with a wound that was almost healed. So, we were trying to figure out how things go* [went] *wrong. What do they do differently to what we are doing here? Patients also complained about the service. (HCP 2, Nurse)* |
| Q5 | *I just wanted to go home. So, every time when I am in the hospital, obviously I just want to go home. I do not like coming to see the doctors. So, even if they explain what is going on with you, you say something like, “Okay, doctor, okay”. You just want to go home; you do not really care about what the doctor says and then you will find out later that maybe you should have listened, or maybe I did not hear properly. It helps if you come with someone who can listen together with you; so, that person who is looking after you can know what is going on as well. It was really helpful when my sister came with me to the hospital so she could also listen to the doctor and what the doctor said.*  *(Patient 4, Specialty A)* |
| Q6 | *Babes* [addressing his partner]*, just fill in the gaps if I am missing stuff here, hey? ... I was concerned because one of the things that Groote Schuur had said was, they want me to cough, because with people who are intubated, they tend to develop pneumonia or TB; what did they say, Babes?*  *(Patient 17, Specialty A)* |
| Q7 | *It was painful; like, how did I get an infection in a hospital? … Anything can go wrong in theatre; your heart can stop. You do not think, “I can get an infection on the operating table” because it is a sterilised area. The doctors work with gloves and all the equipment is sterilised prior to the op.*  *(Patient 5, Specialty B)* |
| Q8 | *I have been around many people that mentioned they got an infection in the hospital, and it has always boggled our minds when we sit around and we talk, how does it happen?*  *(Patient 10, Specialty A)* |
| Q9 | *I have been aware of people becoming infected even within the hospitals, whereas we know our hospitals are* [considered to be] *a place of hygiene, you know, they are clean, especially here. I mean, I have seen the way they clean the hospital while I am here. I have been here so long; so, I have seen how they come and clean the wards and clean the cupboards and everything …*  *(Patient 10, Specialty A)* |
| Q10 | *My question is just, “Why must you get an infection?” That is my question, why? Is it because there is something wrong with your body? Why do you get an infection, especially when you went through surgery? Why? Why do I have to get an infection?*  *(Patient 14, Specialty A)* |
| Q11 | *I was so scared; you must remember, I have got an infection already. So, it works on your nerves; you must sanitise everything and where you sit or where you go. The environment must be clean because you do not want to pick up any germs. Because I do not know how I picked ... was it a germ that I picked up, or did it just come because of the surgery?*  *(Patient 14, Specialty A)* |
| Q12 | *I was not spoken to about an infection that could come in, but I think when you come to hospital you do not come thinking that maybe this is going to go wrong. I think that is why I was so disappointed that I had to go back to the theatre; because, in my mind I was recovering, you see? So, at what point do you tell a patient about infection? People handle things differently. Some people do not want to know all the negative things that may happen, and some doctors may not tell them all these things because it will put them off. I do not know? I cannot say what goes on in their minds and their thoughts, but for myself, I did not feel bad that I was not told about it … (Patient 10, Specialty A)* |
| Q13 | *My honest opinion, I do not think people will go for surgery if they know they can get an infection. They will not, they will never go there, because infection is not nice.*  *(Patient 14, Specialty A)* |
| Q14 | *The number of people that move back and forth* [in that hospital] *was a little bit overwhelming for me. Some of them came in for some serious surgery, others for minor surgery, but I felt the risk of me getting an infection was high because of all these people coming in for all sorts of surgical procedures; psychologically, I was concerned …*  *(Patient 17, Specialty A)* |
| Q15 | *They told me I should breastfeed, and I told the doctor I am not interested in breastfeeding, I do not even feel like it. You know, some days I miss my child, but some days, I am just not interested. I really miss the first one. This second one, I did not know that well, I just remember her because I saw her for a few days and then I was hospitalised …*  *(Patient 4, Specialty A)* |
| Q16 | *It hurts me; I cannot mix with other people, I cannot attend gym as usual, I cannot participate in other social activities.*  *(Patient 19,Specialty A)* |
| Q17 | *How do you treat the depression that someone might go through in this? Because, as I said, I have had bad days. I have got an amazing support system; so, I am lucky, but not everyone has that support system.*  *(Patient 15, Specialty A)* |
| Q18 | *If the patients do get infections, let them go into counselling, implement that. Say, “Okay, you came today; but, if it is possible, can you go for counselling, if it is a correct time for you?” Because mentally, it is traumatising. Mentally, physically, it is traumatising.*  *(Patient 14, Specialty A)* |
| Q19 | *After they removed my womb, I asked the doctor if they can find me a therapist, or someone I can talk to. They agreed and they told me that a counsellor will come, but no one came. All they did, they just came and told me I should breastfeed.*  *(Patient 4, Specialty A)* |
| Q20 | *Communication with the physio we worked with was not {ONLY] during the visiting hours; they were in the area, and they communicated at the same time. The nice part of that was, I could do things with her and tell her, “No, you cannot do that” because I was part of the conversation.*  *(Patient 1, Specialty C)* |
| Q21 | *That is why we like, love, not like, love Dr. M because she will ask you, “Why do you think so?” She comes on your level even though she’s a doctor…*  *(Patient 3, Specialty A)* |
| Q22 | *What I loved about the doctors is that they did not give up on me. As I already explained, they asked people to come and speak to me if I refused to listen. They kept taking me to scans almost every day when I was in the hospital. I think they cared. That is why I told my family I cannot sue the hospital.*  *(Patient 4, Specialty A)* |
| Q23 | *What happened is it got infected. I went to my GP, because I thought, “I do not want to sit in a queue for a day,” and he took one look at it, and said, “I think you had better go to Groote Schuur now.” I came to emergency. I had to wait, but the following day I was put into a ward immediately … The ward was extraordinary. Nurses were amazing. You know, you keep on hearing about how the nurses do not care, and they are blah, blah. No, they were amazing.*  *(Patient 15, Specialty A)* |
| Q24 | *Speaking up is not the problem; it is being listened to that is the problem. I find it a lot that we get dismissed because we are not professionals in this field. It is like you do not know what you are talking about; you can just wait.*  *(Patient 8, Specialty B)* |
| Q25 | *Because I knew I was in the right place. They knew what they were doing … If someone comes*[came] *to me, and it is*[was] *my job, obviously, I will*[would] *do the best, and I was not expecting to ask* [them]*; just letting them do what they are*[were] *best for* [sic]*.*  *(Patient 16, Specialty A)* |
| Q26 | *I was talking with the sister, and I said, “The way you are doing your things and the way the clinic is with my leg is different. So, please, sister, I can’t say to doctor again, that I want to come here every day (for my wound dressing).” The sister said, “Yes, you are free to say it.”*  *(Patient 6, Specialty A)* |
| Q27 | *I used to speak to my aunt. She is the one who told me, “You must ask questions; you need to ask, because it is your foot; so, you need to ask how damaged it is, what are they thinking of doing, what is the plan?” She told me something about the nerves; I do not know how she knew that. I only asked that question on Thursday, I think. And then they said, “That is to check if the nerves are bad,” and if they need to do any procedure.*  *(Patient 16, Specialty A)* |
| Q28 | *They asked if I had any disease; I told them I once had TB, and was on treatment for six months. They asked if I’m smoking, and I said, “No; I used to and then I stopped.” … They told me not to eat or drink any liquid. I asked the doctor what was going to happen, and he said “I’m going to see in* [the] *theatre”.*  *(Patient 19, Specialty A)* |
| Q29 | *I was just mad at everybody in this hospital at that time, as I could do nothing for myself. The only thing I could do was make* [a] *noise with my mouth. Furthermore, I was very sick. So, do not come to me and tell me this and that. Get me better; get me the way I was when I came here; that was all I told them.*  *(Patient 12, Specialty B)* |
| Q30 | *I can speak to them, but I just do not … I just feel like they could have done a better job, you see; they did not need to even do a surgery, that is what I think, but I do not know; they are the doctors.*  *(Patient 7, Specialty B)* |
| Q31 | *They speak to themselves, not to you, and I know when I was in the ward, I asked them, “You are speaking amongst yourselves, but you are not telling me, the patient, what is happening.”*  *(Patient 11, Specialty A)* |
| Q32 | *How am I going to be treated when I am sitting there, and I am helpless? Are my wounds going to be taken care of the way they should be? Am I going to walk out of here with a full*[sic] *leg? This is why I had to delay it because I am not mentally prepared for this.*  *(Patient 8, Specialty B)* |
| Q33 | *We are mediators; we need to interact with the patient. As nurses, we come in because we are the advocate for the patient.*  *(HCP 1 & 2, Nurses)* |
| Q34 | *It* [patient engagement] *is part of the process and very important. The most time that I spend with the patient, engaging with the patient on a one-to-one basis, is in my pre-operative clinic; I am sure that is the longest period that we actually spend with the patient before we do anything but along the journey, we continue communicating with the patient . . . If a patient does not want to hear anything pre-op, there is no way we can consent that patient, because he has to be involved. (HCP 7 & 8, Doctors)* |
